# In-Situ Metabolic Profiling of Different Kinds of *Rheum palmatum* L. by Laser Desorption–Dielectric Barrier Discharge Ionization Mass Spectrometry Imaging

**DOI:** 10.3390/metabo14030131

**Published:** 2024-02-21

**Authors:** Xue Xiao, Xiaokang Guan, Zhouyi Xu, Qiao Lu

**Affiliations:** 1 Department of Laboratory Medicine, Taihe Hospital, Hubei University of Medicine, Shiyan 442000, China; xiaoxue@hbmu.edu.cn; 2 Hubei Key Laboratory of Wudang Local Chinese Medicine Research, Hubei University of Medicine, Shiyan 442000, China; 3 Discipline of Intelligent Instruments and Equipment, Xiamen University, Xiamen 361005, China; 20520220156671@stu.xmu.edu.cn; 4 Pen-Tung Sah Institute of Micro-Nano Science & Technology, Xiamen University, Xiamen 361005, China

**Keywords:** in-situ metabolic profiling, laser desorption, dielectric barrier discharge ionization, mass spectrometry imaging

## Abstract

With its high resolving power and sensitivity, mass spectrometry is considered the most informative technique for metabolite qualitation and quantification in the plant sciences. However, the spatial location information, which is crucial for the exploration of plant physiological mechanisms, is lost. Mass spectrometry imaging (MSI) is able to visualize the spatial distribution of a large number of metabolites from the complex sample surface in a single experiment. In this paper, a flexible and low-cost laser desorption–dielectric barrier discharge ionization-MSI (LD-DBDI-MSI) platform was constructed by combining an LD system with an in-line DBDI source, a high-precision sample translation stage, and an ambient mass spectrometer. It can be operated at a spatial resolution of 20 μm in an atmospheric environment and requires minimal sample preparation. This study presents images of in-situ metabolic profiling of two kinds of plants from different origins, a wild and a farmed *Rheum palmatum* L. From the screen of these two root sections, the wild one presented five more endogenous molecules than the farmed one, which provides information about the differences in metabolomics.

## 1. Introduction

As natural experts in organic synthesis, plants are able to generate large numbers of specific metabolites with widely varying structures, such as lipids, nucleic acids, amino acids, peptides, carbohydrates, organic acids, ketones, aldehydes, amines, steroids, vitamins, signaling molecules, hormones, polyphenols, and some other small-molecule metabolic intermediates, which play an important role in the growth and development of plants [1]. Metabolomic profiling can provide botanists with the chemical fingerprint that represents the biochemical state of an organism or sample at a specific point in time, thus revealing biochemical and molecular mechanisms more effectively [2]. 

In the study of plant metabolomics, chromatography, mass spectrometry (MS), nuclear magnetic resonance spectroscopy, capillary electrophoresis, and infrared spectroscopy have allowed the detailed characterization of metabolites [3]. Among them, MS is considered the most informative technique for component identification and has been widely adopted in plant sciences for its high resolving power and sensitivity [4], such as gas chromatography-MS (GC-MS) and liquid chromatography-MS (LC-MS). Zumsteg et al. used reversed-phase ultrahigh-performance LC coupled with electrospray ionization quadrupole time-of-flight (TOF) MS or triple-quadrupole mass spectrometry (TQ MS/MS) for comparison of nocturnal and diurnal metabolomes; they compared the accurate mass spectra with those in databases for metabolite annotation [5]. Chevalier et al. developed a strategy to locate the fluorescence of prenylated anthranoid in living plant cells and discriminate it from that of the other metabolites; they combined spectral imaging, confocal microscopy, and non-targeted metabolomics using mass spectrometry [6].

However, MS can only provide qualitative and quantitative results for metabolites. The spatial distribution of metabolites in the plant, which is vital for fully resolving the synthesis, regulation, and genetic basis of metabolites and the exploration of plant physiological mechanisms, cannot be obtained in traditional metabolomic studies by MS analysis [7]. In addition, traditional LC-MS or GC-MS is time-consuming. As we all know, MSI is a powerful analytical method that is able to precisely visualize the spatial distribution of a large number of compounds from the complex sample surface in a single experiment with high spatial resolution [8,9]. Generally, spatial metabolomics based on MSI technology obtains information about metabolites in biological samples by scanning biological tissue section samples point by point using MS [10]. Recent decades have witnessed a significant breakthrough in plant analysis brought by mass spectrometry imaging (MSI) because it can determine both the molecular compositions and relative concentrations and spatial distributions, facilitating a comprehensive understanding of the functions and regulation pathways of specific components in plants [11]. 

Matrix-assisted laser desorption/ionization (MALDI)-MSI is now widely used in spatial metabolomics. Hong et al. took it as a tool for identifying food authenticity; a wild or farmed species can be clearly identified through screening the metabolites [12]. Qin et al. mapped the peptide biomarkers in castor beans, revealing a valuable classification clue concerning nationality and altitude [13]. In an attempt to release the sample from the high vacuum to ambient conditions, several atmospheric pressure ionization sources have been developed; they allow the analysis of samples with minimal to no sample workup [14]. Liao et al. visualized the spatial distribution of specialized metabolites in tea (*Camellia sinensis*) using desorption electrospray ionization imaging mass spectrometry [15]. Li et al. mapped the spatial distribution of endogenous molecules in coffee beans by atmospheric pressure MALDI-MSI, and the results demonstrated that it may be used for coffee bean quality identification, efficient use, product traceability, and product counterfeiting [16]. However, the number of neutrals is far greater than the number of ions emitted upon laser irradiation; only one in about one million analytes ejected by laser desorption/ablation is ionized [17]. Thus, the post-ionization strategy, which can enable the ionization of desorbed neutrals to enhance mass spectrometric detection schemes, meets the quest. Nie et al. introduced a vacuum-ultraviolet laser to post-ionize the laser-desorbed materials at the base of MALDI. They visualized the distribution of curcumin in the turmeric root at different maturity periods, but the sample must be put in the high vacuum chamber [18]. Meng et al. developed a 5 μm-resolution laser ablation electrospray ionization-MSI by using a microlensed fiber and visualized metabolites in a parsnip root section [19]. Feng et al. presented the ambient MSI for *Radix Scutellariae* using plasma-assisted laser desorption ionization mass spectrometry with a resolution of 60 μm [20]. Our former work also used plasma to pot-ionize laser-ablated materials, and 20 μm resolution was achieved for the imaging of dosed zebra fish [21].

In this paper, a flexible and low-cost MSI platform was constructed by combining a laser desorption system with an ambient mass spectrometer that was equipped with an in-line dielectric barrier discharge source for post-ionization of laser desorbed neutrals, i.e., laser desorption–dielectric barrier discharge ionization-MSI (LD-DBDI-MSI). Utilizing this MSI platform, we conducted in-situ metabolic profiling of herbal medicines in a wild and a farmed *Rheum palmatum* L., achieving a spatial resolution of 20 μm at atmospheric pressure. The MSI images show that our LD-DBDI-MSI data could provide information about the differences between these two kinds of plants from various origins in metabolomics.

## 2. Materials and Methods

### 2.1. Reagents and Plant Root Slice Preparation 

**Figure 1:**
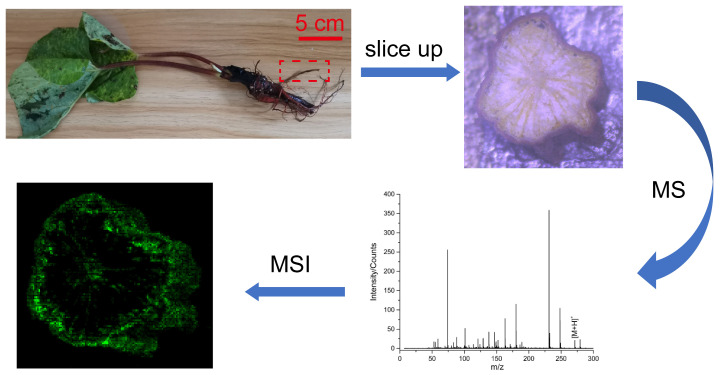
Schematic of the procedures of the whole experiment.

Emodin-3-methyl ether (C_16_H_12_O_5_; MW = 284.26) was purchased from Sigma-Aldrich (St. Louis, MO, USA) and used without further purification. Two kinds of half-year-old *Rheum palmatum* L., which are Rheum species of plants of the *Polygonaceae* family, were under investigation in this study. They were obtained from a farmer in Hanzhong City, Shaanxi Province, China. Plant A represents the wild variety, while plant B is the farmed one under care in a cropland. In this study, sections of the roots of these two plants with a thickness of ~1 mm were manually cut by a blade. To maintain a flat shape during the drying process, these sections were placed in the middle of two glass slides to maintain a flat shape when they were under dry air; otherwise, the slices would be curly. Then the dried sections were mounted to the sample stage for LD-DBDI-MSI analysis. A schematic of the procedures for the whole experiment is shown in Figure 1.

### 2.2. Laser Desorption System

The second harmonic (532 nm) of a Nd:YAG laser (Dawa 100, Beamtech Optronics Co., Ltd., Beijing, China) with a maximum repetition rate of 20 Hz, a pulse duration of 8 ns, a beam divergence of less than 1 mrad, and a beam diameter of 5 mm was used as the laser source. To achieve the required laser irradiance, a changeable filter attenuator was incorporated, and the energy of the laser pulse irradiated onto the sample was measured using an energy measurement meter (Ophir Optronics Solutions LTD, Jerusalem, Israel). In this laser desorption system, a 10× beam expander was introduced to expand the laser beam, and an aperture with an opening of 2 mm diameter was used to suppress the periphery of the expanded laser beam. This configuration enabled the attainment of a smaller diffraction-limiting beam waist. Finally, a lens with a focal length of 50 mm was employed to focus the laser beam onto the sample surface. Additionally, an observation system consisting of a color charge-coupled device (CCD) camera and a variable-focus lens was constructed to monitor the laser sampling processes and capture the optical images of sections. 

### 2.3. Dielectric Barrier Discharge Ionization Source

In this experiment, a plasma ionization source known as dielectric barrier discharge ionization (DBDI) was employed to post-ionize the desorbed materials. This custom-made source was configured based on our previous work [22]. In this configuration, a quartz glass capillary (i.d. = 1.15 mm, o.d. = 1.55 mm, and L = 35 mm) served as the dielectric between a high-voltage electrode and a ground electrode. The high-voltage electrode was an 8 mm-long stainless-steel ring (i.d. = 1.6 mm and o.d. = 3.0 mm), and it must be wrapped tightly around the quartz glass capillary. The ground electrode, which also served as the sampling capillary, was a stainless steel capillary (i.d. = 0.6 mm, o.d. = 1.0 mm, and L = 15 mm) inserted into the quartz glass capillary. The end of this capillary was polished to a cone shape, allowing it to be positioned in close proximity to the point of desorption, thereby enhancing the transmission efficiency of molecules from the laser desorption point to the sampling capillary. 

The quartz glass capillary was connected to the mass spectrometer (MS) using a Teflon inlet, enabling easy interfacing with any MS system equipped with an atmospheric pressure interface. Thus, these four components, along with the MS inlet, were arranged coaxially. This “in-line geometry” design resulted in higher ion transmission efficiency compared to the conventional “open geometry” [23]. Due to the constant under-pressure inside the MS, air could serve as the carrier gas with a fixed flow rate through the capillary (900 mL/min), eliminating the need for specialized auxiliary gas to operate this source. Plasma was ignited inside the capillary by applying an alternating high voltage to the two electrodes, thereby ionizing the gaseous sample molecules carried by the air. In this experiment, the alternating high voltage was set at approximately ~3.8 kV/40 kHz. The electrodes were covered with insulation glue to ensure operator and instrument safety by insulating them from the high-voltage source.

### 2.4. Mass Spectrometry and Mass Spectrometry Imaging Setup

The mass spectrometer employed for ion characterization in this research was a quadrupole/time-of-flight mass spectrometry (Q-TOF MS) system (API-TOF, TOFWERK AG., Thun, Switzerland). The voltage and temperature of the MS inlet capillary were set at 0 V and 200 °C, respectively. The TOF extraction rate was set at 16 kHz. Data acquisition was performed using a mass window of *m*/*z* 7 to 600, and all experiments were conducted in positive ion mode. 

**Figure 2:**
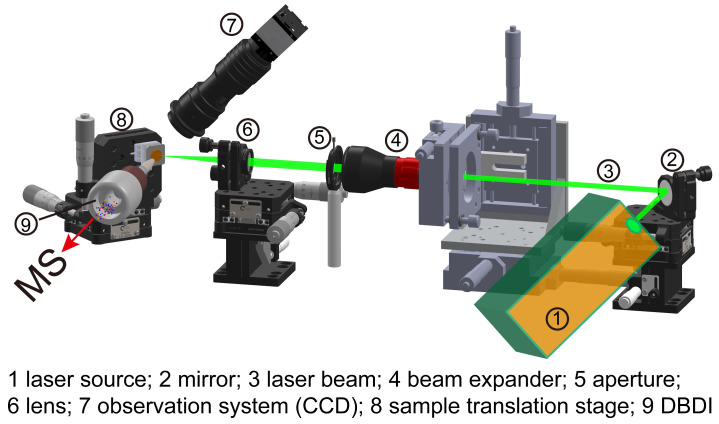
Schematic diagram of the laser desorption–dielectric barrier discharge ionization mass spectrometry imaging apparatus.

For mass spectrometry imaging setup, double-sided tape was used to mount the sample section onto a two-dimensional nanometer precision linear positioner (SLC-1750s, SmarAct GmbH, Oldenburg, Germany), which was used for sample translation. The movement of the sample positioner was synchronized with laser pulse triggering, and the mass spectrometer’s data acquisition card has been described in detail in our previous work [24]. Herein, the translational speed of the sample platform was set at 200 μm/s, and the laser repetition rate was 10 Hz. Each data point on the section was irradiated with a single laser shot, followed by the recording of a mass spectrum. The whole system for the LD-DBDI-MSI experiment contains a laser desorption system, observation system, DBDI source, sample translation system, and MS, which are described in detail in Figure 2.

### 2.5. Data Processing and Drawing

Tofware version 3.2.1 software (TOFWERK AG., Thun, Switzerland) was employed for original data post-processing. The data for mass spectra were processed using OriginPro 2016 (OriginLab, Northampton, MA, USA). The coordinates of each laser desorbed point (x, y) and the intensity of the peak (z) in the mass spectrum extracted from MS were used to generate the data for an MSI image (x, y, z). MSI images were created using commercial scientific graphics software (Surfer 12, Golden Software LLC, Golden, CO, USA).

## 3. Results

### 3.1. Spatial Resolution of MSI

**Figure 3:**
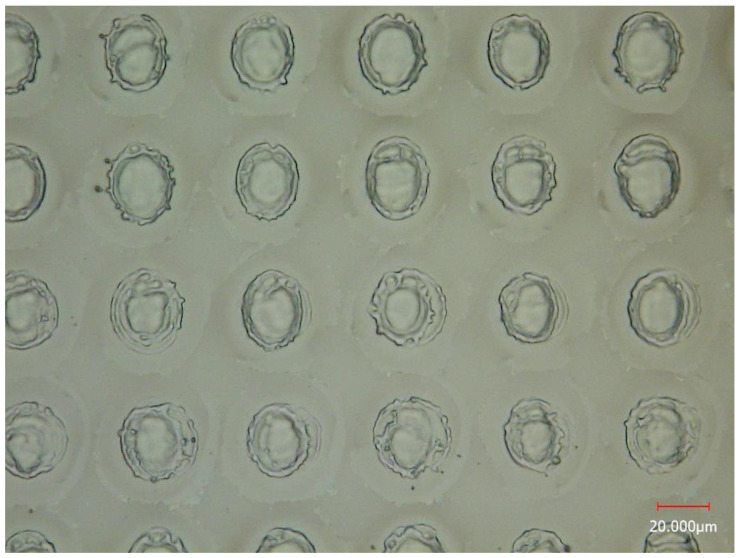
Optical microscopy image of craters on a silicon wafer after laser desorption.

The concept of resolution for MSI is a topic of extensive discussion [25]. In laser-based MSI, the spatial resolution primarily depends on the laser spot size. Consequently, numerous studies define the spatial resolution directly as the diameter of the craters when the step size matches said diameter [26,27]. However, alternative research defines imaging resolution by measuring the distance between the intensities of 16% and 84% in a line scan across the sharp edge of a standard grid sample [28]. In our experiment, the sample translation stage continuously moved forward, shifting vertically at the end of each line scan, and then moved back in preparation for the subsequent line scan. To characterize the spatial resolution of our method, we set the step size (the distance between two craters) at 40 μm. Figure 3 showcases the optical microscopy image of craters on a silicon wafer after laser desorption. It is evident that the craters have a diameter of approximately 20 μm, indicating that the spatial resolution of our MSI experiments is ≈20 μm, according to this definition.

### 3.2. Direct Analysis of Standard Sample by LD-DBDI-MS

**Figure 4:**
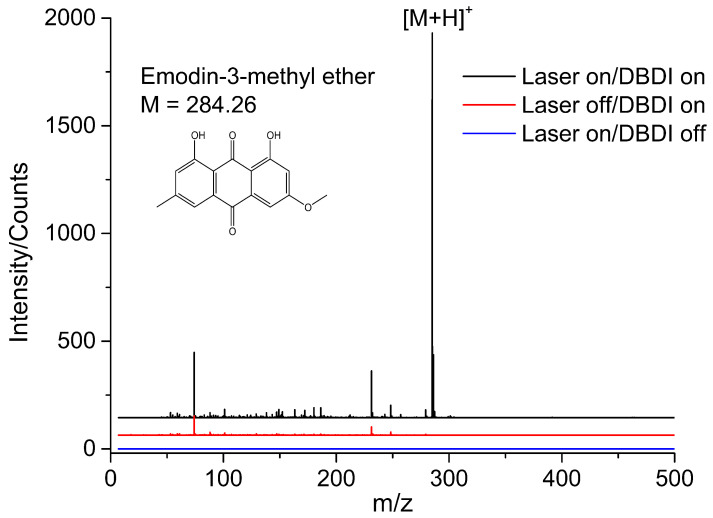
Mass spectrum of emodin-3-methyl ether obtained by laser desorption–dielectric barrier discharge ionization mass spectrometry.

Figure 4 presents the LD-DBDI-MS spectra of standard emodin-3-methyl ether, which was compressed into a pellet using a tablet press and coated onto the sample plate. Initially, the sample was vaporized by the desorption laser, leading to the formation of a gaseous plume above the sample pellet. Subsequently, the gaseous sample molecules, along with air, were drawn into the DBDI source due to the under-pressure within the MS. The species were then ionized by the DBD plasma and detected by the MS. The blue trace in Figure 4 reveals that no distinct mass spectral signals were observed when the ionization source was deactivated, even though the desorption laser was triggered. This indicates that a single desorption laser, without post-ionization, fails to generate a meaningful signal contribution in this experiment. The red trace displaced the mass spectrum when only the DBDI source was turned on; therefore, it represents the background signals from lab air, such as plasticizers and volatile reagents [22]. The black trace illustrates the mass spectrum when both the desorption laser and DBDI source were activated, revealing the characteristic peak of emodin-3methyl ether (*m*/*z* 285) in the spectrum. 

### 3.3. Identification and Distribution of Endogenous Molecules from Different Rheum palmatum L.

**Figure 5:**
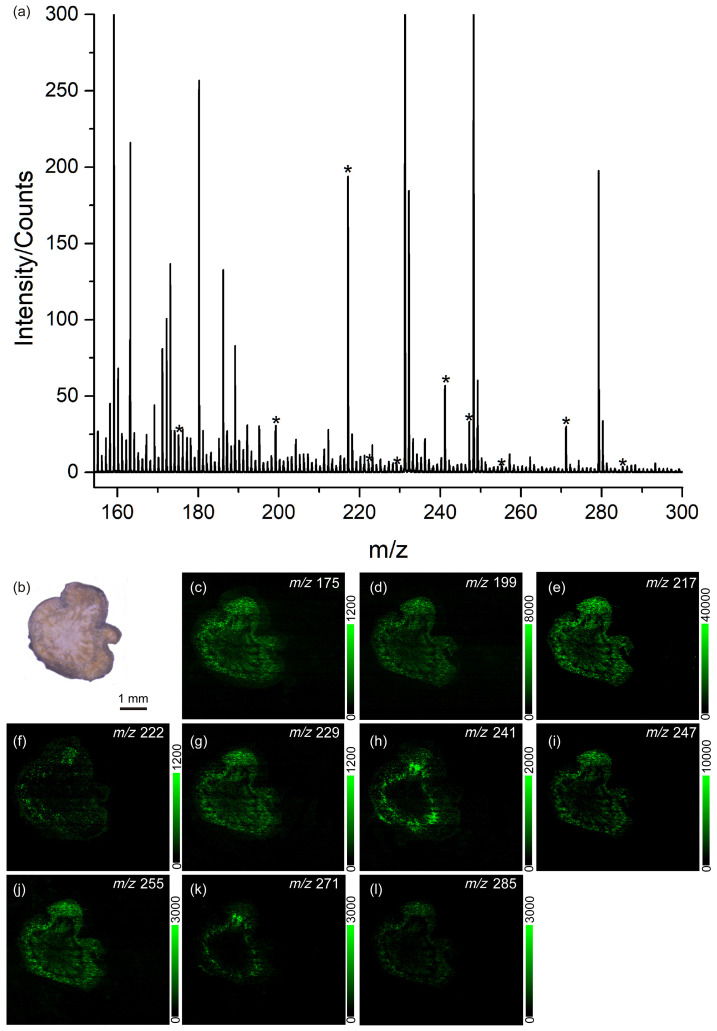
(**a**) Mass spectrum of *Rheum palmatum* L. (Plant A) obtained by laser desorption–dielectric barrier discharge ionization mass spectrometry. (**b**) Optical microscopy image of the root section (**c**–**l**) LD-DBDI-MSI of different selected ions at (**c**) *m*/*z* 175, (**d**) *m*/*z* 199, (**e**) *m*/*z* 217, (**f**) *m*/*z* 222, (**g**) *m*/*z* 229, (**h**) *m*/*z* 241, (**i**) *m*/*z* 247, (**j**) *m*/*z* 255, (**k**) *m*/*z* 271, and (**l**) *m*/*z* 2885. The peaks marked with asterisks at *m*/*z* 175, *m*/*z* 199, *m*/*z* 217, *m*/*z* 222, *m*/*z* 229, *m*/*z* 241, *m*/*z* 247, *m*/*z* 255, *m*/*z* 271, and *m*/*z* 285 correspond to endogenous species.

In order to identify the endogenous species, a thin slice was excised from the root of *Rheum palmatum* L. (Plant A) and subjected to LD-DBDI-MS analysis without complex sample pretreatment. The whole mass spectrum with a mass range of 0~400 was shown in Figure S1. To make the peaks of the endogenous species more clear, a zoomed spectrum with a mass range of 160~300 is shown here. Figure 5a displays the MS spectrum of this section, with random sampling conducted on its surface. The peaks marked with asterisks at *m*/*z* 175, *m*/*z* 199, *m*/*z* 217, *m*/*z* 222, *m*/*z* 229, *m*/*z* 241, *m*/*z* 247, *m*/*z* 255, *m*/*z* 271, and *m*/*z* 285 correspond to endogenous species. Unfortunately, due to the absence of MS/MS capabilities in our mass spectrometer, only five kinds of endogenous molecules, *m*/*z* 241 [C_14_H_8_O_4_+H]^+^, *m*/*z* 247 [C_14_H_14_O_4_+H]^+^, *m*/*z* 257 [C_14_H_8_O_5_+H]^+^, *m*/*z* 271 [C_15_H_10_O_5_+H]^+^, and *m*/*z* 285 [C_15_H_8_O_6_+H]^+^, have been identified by comparison with mass spectra of standard samples and the literature [29]. The unlabeled peaks represent background signals, similar to the red trace in Figure 4. Figure 5b shows the optical microscopy image of this section, which was captured by the observation system. In an attempt to investigate the localization and relative concentration of the above endogenous molecules in the root of Plant A, LD-DBDI-MSI experiments were performed with a scanned area of 6 × 6 mm^2^ and a step size of 20 μm. Figure 5c–l shows the MSI images of this section for the *m*/*z* values of (c) 175, (d) 199, (e) 217, (f) 222, (g) 229, (h) 241, (i) 247, (j) 255, (k) 271, and (l) 285, respectively. The value of the green scales in Figure 5c–l is calculated by integrating the area of each mass peak. From the scale, the relative concentration of the species in the section can be clearly observed.

**Figure 6:**
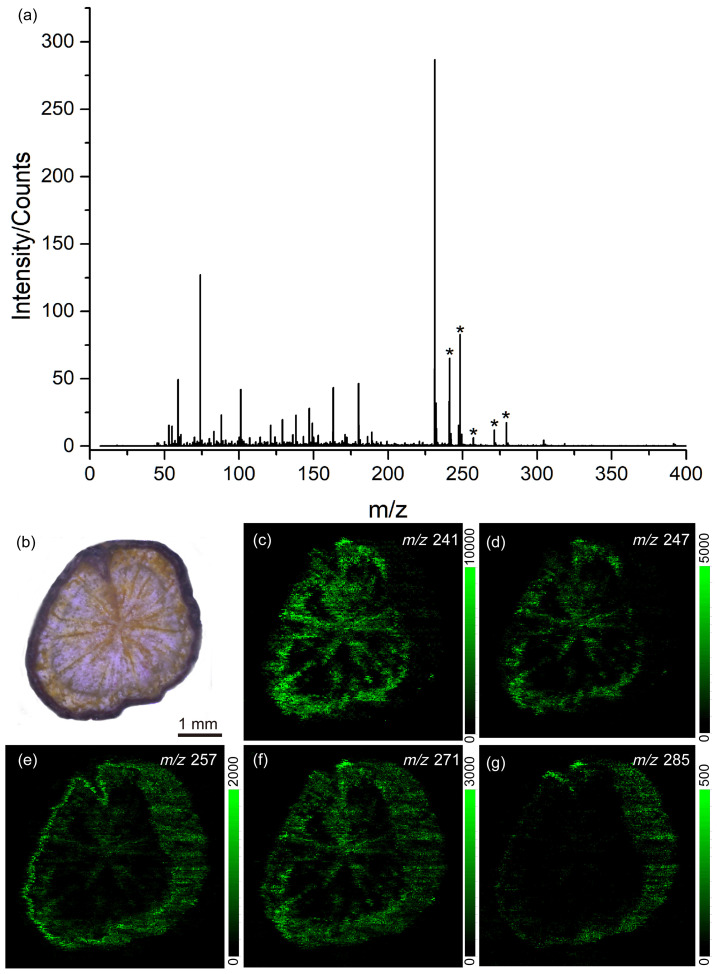
(**a**) Mass spectrum of *Rheum palmatum* L. (Plant B) obtained by laser desorption–dielectric barrier discharge ionization mass spectrometry. (**b**) Optical microscopy image of the root section (**c**–**g**) LD-DBDI-MSI of different selected ions at (**c**) *m*/*z* 241, (**d**) *m*/*z* 247, (**e**) *m*/*z* 257, (**f**) *m*/*z* 271, and (**g**) *m*/*z* 285. The peaks marked with asterisks at *m*/*z* 241, *m*/*z* 247, *m*/*z* 255, *m*/*z* 271, and *m*/*z* 285 correspond to endogenous species.

To discern the differences in endogenous species among different plants, we obtained a thin slice from the root of *Rheum palmatum* L. (Plant B) and subjected it to MS analysis, following the same procedure as above. Figure 6a displays the LD-DBDI-MS spectrum of this section, whose optical microscopy image is shown in Figure 6b. The peaks marked with asterisks at *m*/*z* 241 [C_14_H_8_O_4_+H]^+^, *m*/*z* 247 [C_14_H_14_O_4_+H]^+^, *m*/*z* 257 [C_14_H_8_O_5_+H]^+^, *m*/*z* 271 [C_15_H_10_O_5_+H]^+^, and *m*/*z* 285 [C_15_H_8_O_6_+H]^+^, were five kinds of endogenous species that have been detected. Figure 6c–g shows the corresponding MSI images, which were obtained by LD-DBDI-MSI. The scanned area is 6 × 6 mm^2^ with a step size of 20 μm.

## 4. Discussion

As we all know, laser ionization efficiency is rather low (<10^−4^) when the laser fluences are less than 1 × 10^8^ W/cm^2^, and most of the laser-ablated materials are neutrals [30,31]. Thus, the MS signal of the analytes is often imperceptible when post-ionization is not employed to enhance the total ionization efficiency, which results in low sensitivity. Because high laser energy may result in larger craters (poorer MSI spatial resolution), it is a good way to lower the laser energy and use a post-ionization method to improve the total ionization efficiency [32]. In this experiment setup, the laser desorption and plasma ionization processes were separated spatially and temporally, allowing for independent optimization of these two steps. From the blue trace in Figure 4, we can see that almost no significant ions could be detected when only the desorption laser was employed on the sample. Surprisingly, after turning on the DBDI source, which has been optimized (alternating high voltage), the specific mass signal of the analyte appeared. With these desorption/ionization step separation strategies, we can optimize the laser energy, wavelength, repetition rate, et al., and choose the appropriate ionization source for molecules with different polarities.

Compared with other ionization methods, such as laser post-ionization with its associated optical system, DBD could potentially serve as a much cheaper and more convenient post-ionization source. It has been widely applied in many fields, such as food safety, environmental monitoring, biochemical analysis, and forensic authentication, since it was first described as an ambient ionization source 10 years ago [33]. In analytical mass spectrometry, some conventional “open geometry” DBDI devices have been coupled to an ambient MS for detecting nonvolatile compounds at ultra-trace level [34]. However, flowing He gas (99.9999%) must be used to generate the plasma. The ionization source used in our experiment is an “in-line geometry” designed DBDI; it can be taken as the extending of the capillary of MS, and He gas can be eliminated [35]. This design ensures a much higher efficiency of ion transfer to the MS, which promises the ability to analyze trace metabolites in analytes [36,37]. 

Mapping the distribution of the endogenetic species, especially the active ingredient, is helpful for botanists to improve their understanding of the relationship between compound distribution and plant structure [38]. In this experiment, the root slices were scanned with a spatial resolution of 20 μm via LD-DBDI-MSI, which is at the single-cell scale for plants. It can be seen from the MSI images in Figure 5 (plant A) that most of the detected species were concentrated at the epidermis of the root. However, when we analyze the distribution of the endogenetic species in plant B (as shown in Figure 6), we find that five species are at the same *m*/*z* as plant A, and most of the detected species are not only concentrated at the epidermis of the root but also at the edge of the vascular cylinder. In addition, we can also observe the edge of its vascular cylinder and wood rays (radial stripes) in xylem clearly, which are both invisible in plant A. The results indicate that the distribution of active ingredients in different sections can be successfully achieved and also provide information about the differences between *Rheum palmatum* L. from various origins. We think it can also improve the efficiency of utilization in the treatment and extraction of active components in industrial production. However, it is a pity that some detected species in plant A have not been characterized, given that the MS we used was a simple quadrupole/time-of-flight mass spectrometry (Q-TOF MS) system rather than a more advanced instrument like an Orbitrap-MS or FT-ICR-MS, and no data base can be provided. It is well known that high-resolution mass analyzers, especially those containing a tandem mass spectrometer, could provide untargeted information for investigative study designs, gathering a wealth of information in physiology, disease, and fundamental research [39,40]. 

## 5. Conclusions

In this paper, we combined a laser desorption system, an automatic X−Y nanometer precision sample translation stage, an in-line dielectric barrier discharge source for post-ionization of laser desorbed neutrals, and an ambient mass spectrometer to construct a low-cost and flexible LD-DBDI-MSI platform. It separated the laser desorption and plasma ionization processes in time and space, which enabled us to optimize these two steps independently. This unique approach allows for the enhancement of spatial resolution in MSI by reducing the energy of laser radiation and improving plasma post-ionization efficiency. This outstanding technology achieves high spatial resolution, high sensitivity, and high throughput, so complex sample pretreatment is not needed. Then, we applied this LD-DBDI-MSI platform to image the distribution of active components in the roots of two kinds of *Rheum palmatum* L. with a lateral resolution of 20 μm. Interestingly, these two plants (wild and farmed, in the same growing area but in different cultivations), represented different endogenetic species and distributions. In conclusion, our methods achieved in-situ metabolic profiling of a wild and a farmed *Rheum palmatum* L. by LD-DBDI-MSI at single cellular spatial resolution.

## Data Availability

The data are available from the corresponding author on reasonable request. The data are not publicly available due to we have no public database and no link to archived datasets analyzed.

## Supplementary Materials

The following supporting information can be downloaded at: https://www.mdpi.com/article/10.3390/metabo14030131/s1, Figure S1: Mass spectrum of *Rheum palmatum* L. (Plant A) obtained by laser desorption-dielectric barrier discharge ionization mass spectrometry.
