# Supplementary material for: In-Situ Metabolic Profiling of Different Kinds of Rheum palmatum L. by Laser Desorption–Dielectric Barrier Discharge Ionization Mass Spectrometry Imaging
Source: Metabolites. 2024 Feb 21;14(3):131. doi: 10.3390/metabo14030131 (PMC10972210; doi:10.3390/metabo14030131)
Supplement: Supplementary file 1 [file metabolites-14-00131-s001.zip › metabolites-2862491-supplementary.pdf]

In-Situ Metabolic Profiling of Different Kinds of *Rheum palmatum L.* by Laser Desorption–Dielectric Barrier Discharge Ionization Mass Spectrometry Imaging

Xue Xiao [1,2], Xiaokang Guan [3], Zhouyi Xu [4,*] and Qiao Lu [1,2,*]

1 Department of Laboratory Medicine, Taihe Hospital, Hubei University of Medicine, Shiyan 442000, China; xiaoxue@hbmu.edu.cn
2 Hubei Key Laboratory of Wudang Local Chinese Medicine Research, Hubei University of Medicine, Shiyan 442000, China
3 Discipline of Intelligent Instruments and Equipment, Xiamen University, Xiamen 361005, China; 20520220156671@stu.xmu.edu.cn
4 Pen-Tung Sah Institute of Micro-Nano Science & Technology, Xiamen University, Xiamen 361005, China
* Correspondence: zhouyixu@xmu.edu.cn (Z.X.); qiaolu@hbmu.edu.cn (Q.L.)

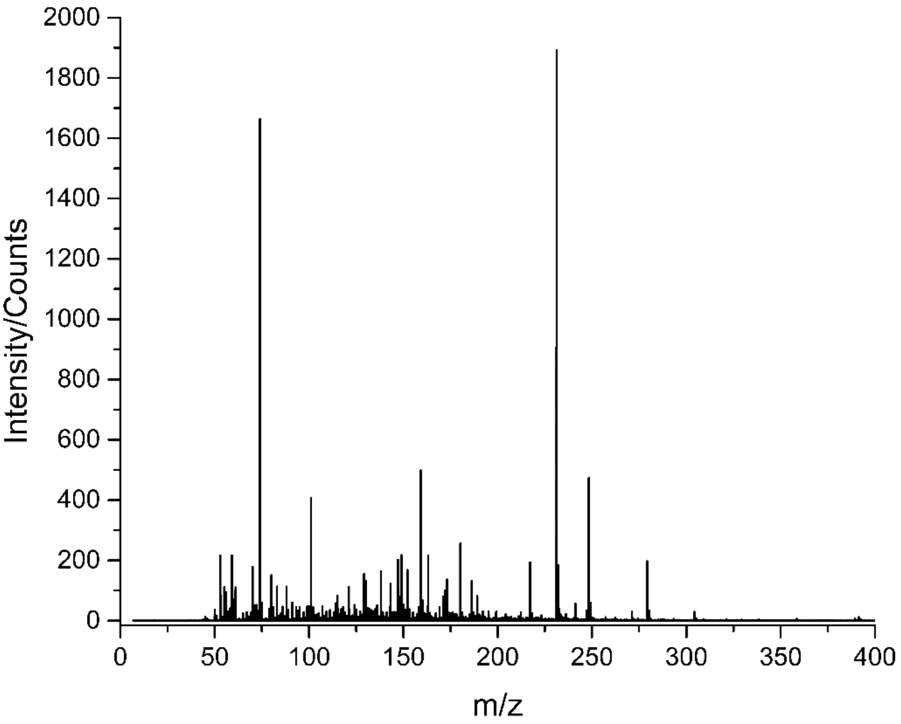

Figure S1. The whole mass spectrum of *Rheum Palmatum L.* (Plant A) obtained by laser desorption-dielectric barrier discharge ionization mass spectrometry.
